# Non-Atherosclerotic Myocardial Infarction With a Presentation of Wellens Syndrome

**DOI:** 10.7759/cureus.9116

**Published:** 2020-07-10

**Authors:** Muhammad B Jamshaid, Aamir Shahzad, Phool Iqbal, Zohaib Yousaf

**Affiliations:** 1 Internal Medicine, Hamad General Hospital, Doha, QAT; 2 Medicine, Hamad General Hospital, Doha, QAT; 3 Internal Medicine, Hamad Medical Corporation, Doha, QAT; 4 Clinical Research, Dresden International University, Dresden, DEU

**Keywords:** wallen syndrome, nstemi

## Abstract

Myocardial infarction (MI) is a critical event that needs timely diagnosis and prompt management. Wellens syndrome can progress to MI if not managed in a timely manner. It implies the underlying critical stenosis of the left anterior descending (LAD) artery of the heart. In this report, we discuss an interesting presentation of pseudo-Wellens syndrome in a hypertensive middle-aged woman admitted as a case of Non-ST-elevation myocardial infarction (NSTEMI). During the hospital stay, she had an episode of chest pain with typical ECG changes, suggesting Wellens syndrome. However, upon intervening with coronary angiography, it turned out to be unremarkable for any coronary artery stenosis. She developed another episode of chest pain during her hospital stay with abnormal ECG patterns requiring further investigations with a non-invasive CT scan of coronary arteries and cardiac MRI for any infiltrative diseases. All workups were unremarkable. A multidisciplinary team involving the medical and interventional cardiology departments were involved in the diagnosis, and the patient was labeled as a case of vasospastic angina. She was treated with calcium channel blockers and was followed up as an outpatient for seven months with no further complications.

Our main objective was to highlight the interesting phenomenon of Wellens and pseudo-Wellens syndrome. The condition requires early diagnosis and timely management to make sure that no underlying critical pathology is present that can result in fatal complications like MI or cardiac arrest.

## Introduction

Wellens syndrome is a group of signs and symptoms characterized by an ECG pattern that shows biphasic T wave or symmetrical T wave inversion in leads V1 to V3 with normal or minimal elevation of cardiac enzymes. The typical ECG changes are known as Wellens sign. It is usually associated with critical stenosis of the proximal left anterior descending (LAD) coronary artery, However, if this sign is seen with normal coronary arteries, then it is referred to as pseudo-Wellens sign [1-4]. We present an interesting case of a middle-aged lady who was admitted for workup of chest pain and developed pseudo-Wellens sign during her hospital stay.

## Case presentation

**Figure 1:**
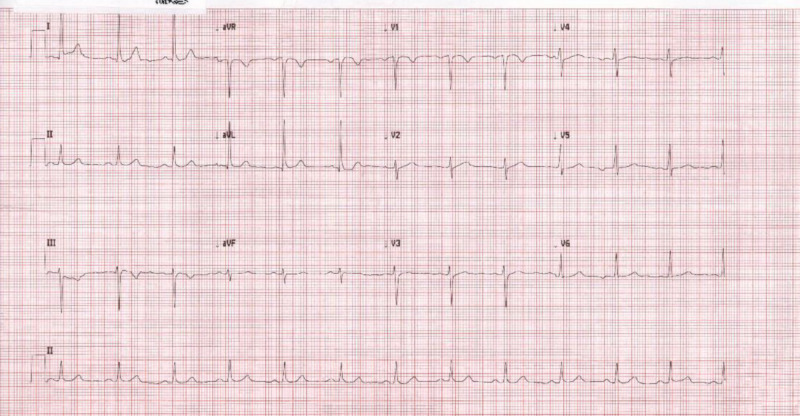
ECG on admission ECG: electrocardiogram

A 42-year-old hypertensive (complaint with medicine), ex-smoker female presented with an episode of chest pain that was acute at the onset, central, excruciating, and pressure-like. There was no associated nausea, vomiting, or dizziness. On investigation, non-ischemic changes on the ECG were observed, as shown in Figure 1, along with elevated cardiac markers: cardiac troponins with a value of 80 ng/L (upper reference limit = 14 ng/L). A working diagnosis of non-ST-elevation myocardial infarction (NSTEMI) was made and the patient's medical management was started. She was initially given a loading dose of aspirin 100 mg and clopidogrel 300 mg, followed by 75 mg of aspirin and clopidogrel respectively. Further plan for coronary angiography with or without angioplasty was made and she was prepared for the same.

**Figure 2:**
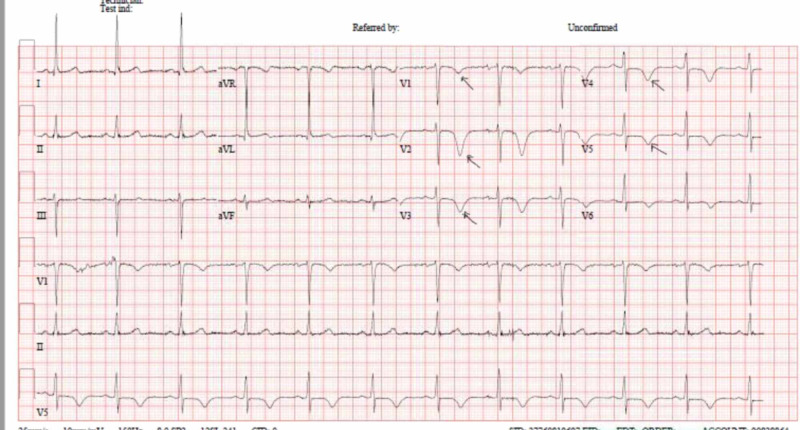
ECG on the second day of hospitalization Symmetric T wave inversion in the chest leads from V1-V6 (Wellens syndrome) ECG: electrocardiogram

The patient remained asymptomatic till day two of hospitalization when she developed another episode of chest pain. ECG showed diffuse symmetrical T wave inversion in precordial leads V1-V6 (Wellens sign) and T inversion in I, augmented Vector Left (aVL) (Figure 2). There was a rise in troponin T from 80 to 215 ng/L. Her chest pain responded to sublingual nitrates, and isosorbide dinitrate (ISDN) infusion was started.

**Figure 3:**
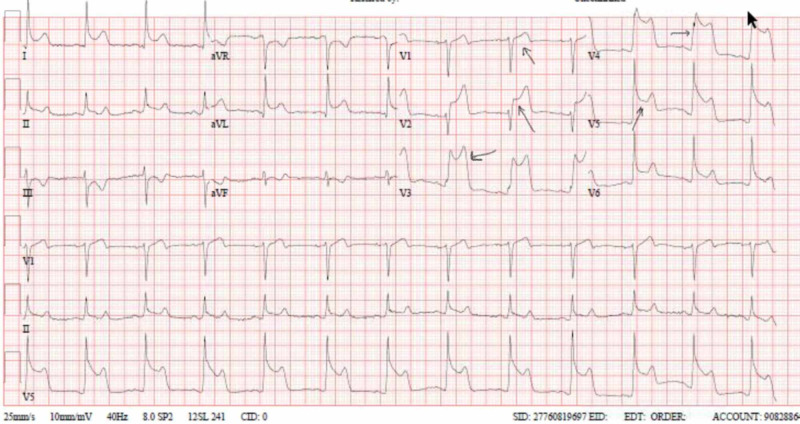
ST elevation in chest leads (V1-V6) and lateral chest lead (I, aVL) aVL: augmented Vector Left

The patient underwent urgent coronary angiography after the second episode of chest pain, which was unremarkable for any coronary artery abnormality. ISDN infusion was stopped and a beta-blocker drug, bisoprolol, was started with a plan to perform a cardiac stress test on an outpatient basis. However, the patient developed another episode of similar chest pain at night, with significant ST-segment elevation in the precordial leads from V1-V6 and in limb leads 1 and aVL as shown in Figure 3. This episode was manged by glyceryl trinitrate (GTN) oral spray. There was no rise in cardiac markers with this third episode. This led to a diagnosis of vasospastic angina, and bisoprolol was stopped. Diltiazem, a calcium channel blocker, was initiated with sublingual GTN as needed for chest pain episodes.

The possibility of an atherosclerotic plaque that could have been missed on coronary angiography was considered, and a repeat coronary angiography was planned. The patient, however, remained asymptomatic and ECG changes reverted to normal. After a multidisciplinary team discussion involving medical and interventional cardiology, it was decided to proceed with a non-invasive CT coronary angiography, which also turned out to be normal. In order to rule out other possible diagnoses like cardiac infiltrative diseases such as sarcoidosis and hemochromatosis, an MRI of the heart was performed, which came back normal as well. The patient remained stable throughout the hospital course with no further complications and was discharged on diltiazem with the diagnosis of vasospastic angina. During her follow-up for seven months as an outpatient, she did not require any hospital admission, and her symptoms were controlled by medical management.

## Discussion

Wellens syndrome is characterized by biphasic T wave or symmetrical T wave inversion in leads V1 to V3. This is a sign of impending MI. Proximal LAD is the typical site of involvement in the form of critical luminal stenosis/plaque. Urgent angiography with angioplasty or coronary artery bypass graft is indicated [5].

Diagnostic criteria for Wellens syndrome are as follows:

· Deeply inverted T waves in leads V2 and V3 OR biphasic T waves in V2

PLUS

· Isoelectric or minimally elevated ST-segment of less than 1 mm

· Preservation of precordial R-wave progression AND no precordial Q waves

· Recent history of angina

· ECG pattern present in a pain-free state

· Normal or slightly elevated cardiac markers [2].

Coronary artery spasm associated with pseudo-Wellens syndrome is characterized by chest pain, usually at rest, which occurs mainly from midnight to early morning. It presents as ST elevation on ECG in the form of the pattern. Triggers inducing chest pain include hyperventilation and beta-blockers. Symptoms usually improve with the administration of calcium channel blockers [6].

Pseudo-Wellens syndrome resulting from coronary artery spasm is rarely reported in the literature; after the resolution of the spasm, coronary flow is restored, which leads to reperfusion injury-related repolarization abnormalities depicted on ECG in precordial leads with inverted or biphasic T waves. Upon recovery of stunned myocardium, T waves are normalized [7]. Pseudo-Wellens syndrome can be associated with pulmonary embolism, right ventricular hypertrophy, and cocaine-induced vasospastic angina.

In this case, the patient had typical features of Wellens syndrome on ECG (deeply inverted T wave inversion V1 and V2). She also had chest pain and normal cardiac enzymes, which fulfilled the criteria of Wellens syndrome (proximal LAD lesion); however, angiography was normal, hence the diagnosis of vasospastic angina was made.

## Conclusions

Recognizing the ECG pattern of Wellens syndrome and differentiating it from pseudo-Wellens is important as the method of treatment varies between the two. Appropriate and timely management of the condition is critical as it may help to avoid the development of MI.
